# *Aspergillus terreus* spondylodiscitis following an abdominal stab wound: a case report

**DOI:** 10.1186/s13256-019-2109-5

**Published:** 2019-06-05

**Authors:** Yasutaka Takagi, Hiroshi Yamada, Hidehumi Ebara, Hiroyuki Hayashi, Satoshi Kidani, Shunro Okamoto, Yuta Nakamura, Yoshiyuki Kitano, Kenji Kagechika, Satoru Demura, Takuro Ueno, Kengo Shimozaki, Hiroyuki Tsuchiya

**Affiliations:** 1 Department of Orthopaedic Surgery, Tonami General Hospital, 1-61 Shintomi-cho, Tonami City, Toyama, 939-1395 Japan; 2 Department of Rehabilitation Medicine, Kanazawa Medical University, 1-1 Daigaku, Uchinada-machi, Kahoku-gun, Ishikawa 920-0293 Japan; 3 Department of Orthopaedic Surgery, Graduate School of Medicine, Kanazawa University, 13-1 Takara-machi, Kanazawa City, Ishikawa 920-8641 Japan

**Keywords:** *Aspergillus terreus*, Fungal infection, Spondylodiscitis, Open fracture of thoracic vertebra, Vertebral osteomyelitis, Antifungals

## Abstract

**Background:**

*Aspergillus terreus*, a saprophytic fungus, is recognized as an emerging pathogen in various infections in humans. However, bone and joint involvement is uncommon. To the best of our knowledge, only seven cases of spondylodiscitis caused by *Aspergillus terreus* have been reported previously in humans. We report a case of a patient with *Aspergillus terreus* spondylodiscitis following an abdominal stab wound.

**Case presentation:**

A 74-year-old Japanese man with no particular medical history fell from a ladder and sustained a left abdominal stab wound from an L-shaped metal peg. Computed tomography showed the trace of the L-shaped metal peg from the left abdomen to the left rib and left kidney. The scan also showed an anterolateral bone avulsion of the left side of the T12 vertebral body, as well as fractures of the L1 left transverse process and the left 10th–12th ribs. He was hospitalized and treated with conservative therapy for 6 weeks. He was readmitted to the hospital with complaints of sudden back pain, numbness of both legs, and inability to walk 13 weeks after the fall. Magnetic resonance imaging findings were typical of spondylodiscitis. Gadolinium-enhanced T1-weighted magnetic resonance imaging indicated increased signal intensity at T11–T12 vertebral bodies and severe cord compression and epidural abscess at T11–T12 associated with infiltration of soft paravertebral tissues. On the seventh day after admission, he underwent partial laminectomy at T11 and posterior fusion at T9 to L2. The result of his blood culture was negative, but *Aspergillus terreus* was isolated from the material of T11–T12 intervertebral disc and vertebral bodies. His *Aspergillus* antigen was positive in a blood examination. Histological examination showed chronic suppurative osteomyelitis. On the 35th day after admission, he underwent anterior fusion at T11 and T12 with a rib bone graft. For 5 months, voriconazole was administered, and he wore a rigid corset. Posterior partial laminectomy at T11 and anterior fusion at T11 and T12 resulted in a good clinical course. The patient’s neurological dysfunction was completely recovered, and his back pain disappeared. Two years after the operation, computed tomography was performed and showed bone fusion at T11 and T12. Magnetic resonance imaging revealed no evidence of increased signal intensity at T11–T12 vertebral bodies and severe cord compression and epidural abscess at T11–T12.

**Conclusions:**

To our knowledge, this is the first report of spondylodiscitis caused by *Aspergillus terreus* after an abdominal penetrating injury. The histological finding of chronic suppurative osteomyelitis and the radiological findings strongly suggested direct inoculation of *Aspergillus terreus*.

## Background

*Aspergillus terreus*, a saprophytic fungus, is recognized as an emerging pathogen in various infections in humans. However, bone and joint involvement is uncommon [1]. Bone aspergillosis is a rare disease, accounting for 1.8% of aspergillosis cases [2]. Molds belonging to the genus *Aspergillus* are important causes of fungal spondylodiscitis. *Aspergillus fumigatus*, *Aspergillus flavus*, and *Aspergillus nidulans* are the most commonly isolated species [3]. There are few reports of vertebral infections involving *A. terreus*. To the best of our knowledge, only seven cases of spondylodiscitis caused by *A. terreus* have been reported previously in humans [1]. We report a case of a patient with *A. terreus* spondylodiscitis following an abdominal stab wound.

## Case presentation

**Fig. 1:**
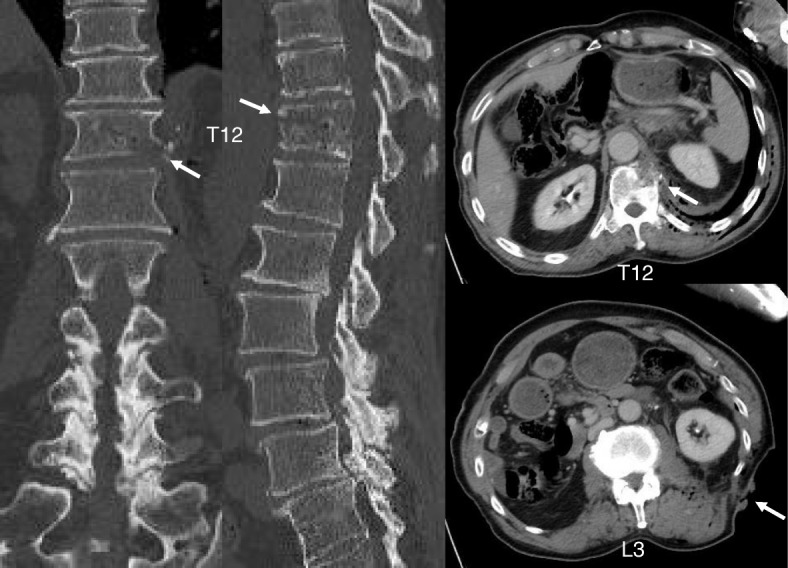
Reconstruction computed tomography showed the fracture of T12 vertebral body. The fracture line passed from the left inferior edge of T12 vertebral body towards the central upper edge of T12 vertebral body (left, middle: fracture line: white arrows). Axial views of computed tomography scan showed the fracture of T12 vertebral body and an abdominal stab wound at L3 level (right upper: anterolateral bone avulsion of the left side of the T12 vertebral body: white arrow, right under: an abdominal stab wound: white arrow)

**Fig. 2:**
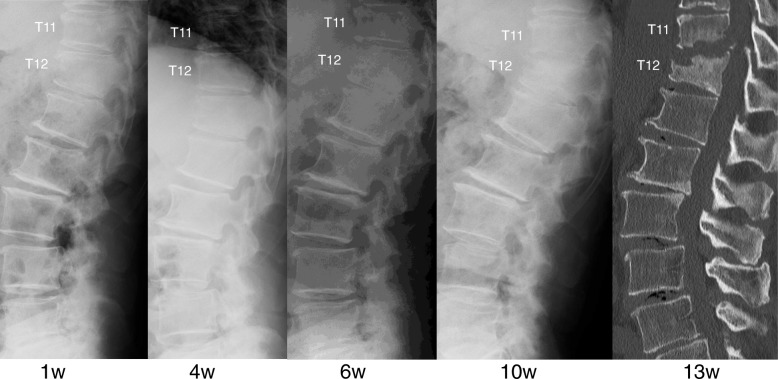
X-ray image showing narrowing of T11–T12 intervertebral disc space at 6 weeks and 10 weeks. A computed tomographic scan at 13 weeks showed numerous irregular osteolytic cavities in T11 and T12 vertebral bodies and destruction of the inferior endplate of T11 and the superior endplate of T12

**Fig. 3:**
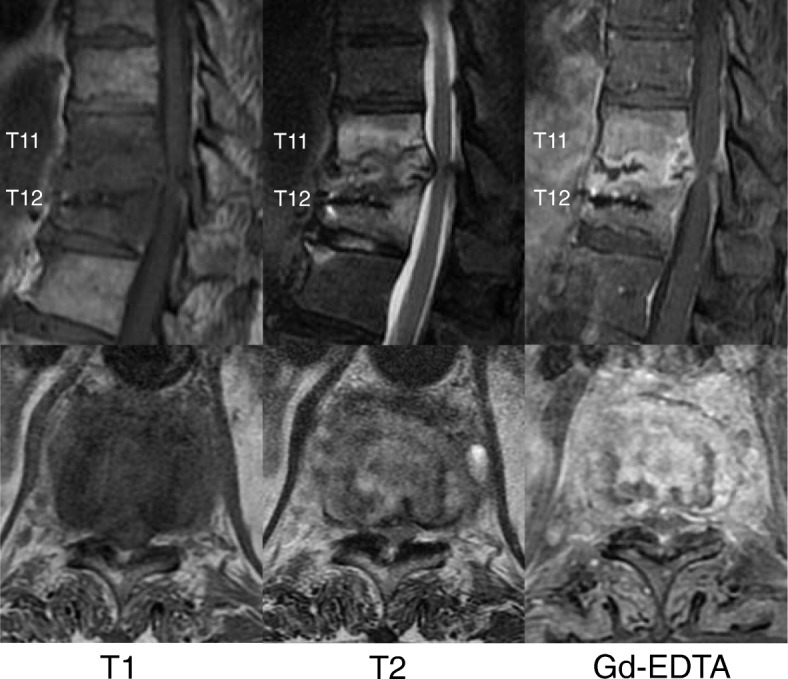
Gadolinium-enhanced T1-weighted magnetic resonance imaging indicated increased signal intensity at T11–T12 vertebral bodies and severe cord compression and epidural abscess at T11–T12 associated with infiltration of soft paravertebral tissues

**Fig. 4:**
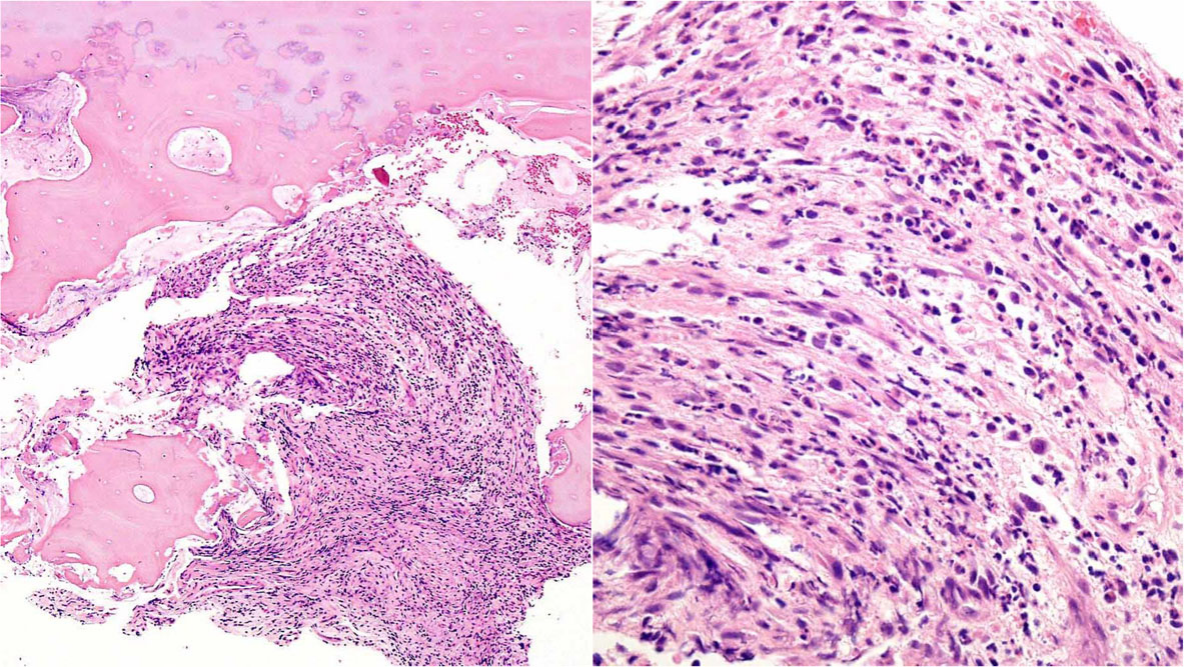
Operative specimen from the T12 vertebra. *Left*: Inflammatory granulation between trabecular bone (H&E stain, original magnification × 100). *Right*: Numerous neutrophils seen in the granulation (H&E stain, original magnification × 400). Histological examination showed chronic suppurative osteomyelitis

**Fig. 5:**
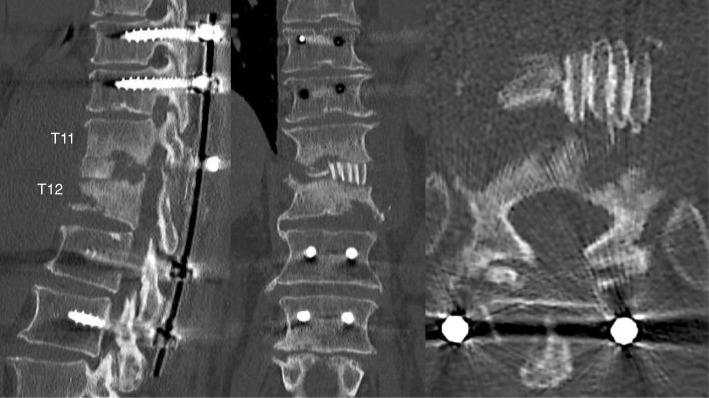
Postoperative computed tomography demonstrated anterior fusion at T11 and T12 with rib bone graft

**Fig. 6:**
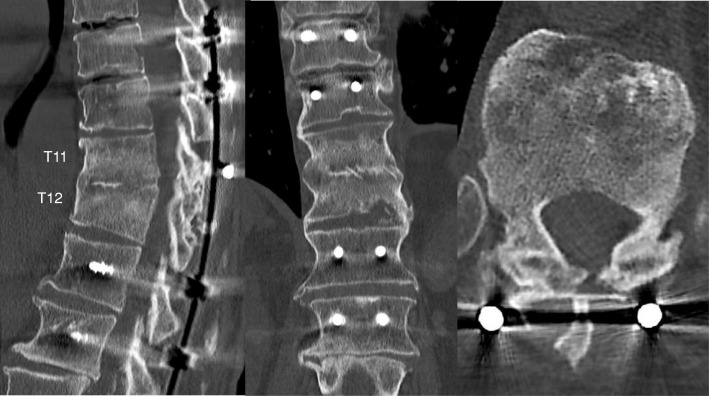
Two years after the operation, computed tomography demonstrated bone fusion at T11 and T12

**Fig. 7:**
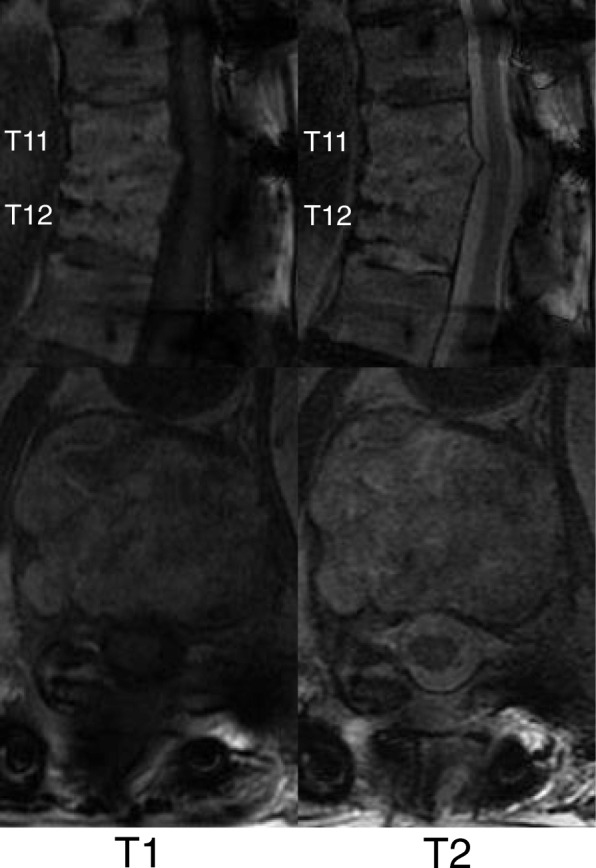
Magnetic resonance imaging revealed no evidence of increased signal intensity at T11–T12 vertebral bodies and severe cord compression and epidural abscess at T11–T12

A 74-year-old Japanese man with no particular medical history fell down from a ladder and sustained a left abdominal stab wound from an L-shaped metal peg on the ground, which he removed by himself. He was brought to the emergency department in our hospital. He had no relevant family or past medical history and no history of smoking or alcohol consumption. Physical examination showed a 5-cm, jagged linear wound on the left abdomen. Computed tomography (CT) showed the trace of the L-shaped metal peg from the left abdomen to the left rib and left kidney, anterolateral bone avulsion of the left T12 vertebral body, and fracture of the L1 left transverse process and the left 10th–12th ribs (Fig. 1). The result of the patient’s neurological examination was normal. Surgical exploration was performed with the patient under local anesthesia in the emergency department, which showed no evidence of peritoneal penetration. The wound was washed with 6 L of physiological saline, and a drain was inserted; the patient was hospitalized for 6 weeks. Ceftriaxone sodium hydrate was administered for 7 days to prevent bacterial infection. One week after the event, the patient started to walk with a rigid corset, and he was discharged in 6 weeks. X-ray images showed narrowing of T11–T12 intervertebral disc space at 6 weeks and 10 weeks. He was readmitted to the hospital with complaints of back pain, numbness of both legs, and inability to walk 13 weeks after the fall. The patient was hospitalized in the orthopedic surgery department. CT showed numerous irregular osteolytic cavities in T11 and T12 vertebral bodies and destruction of the inferior endplate of T11 and the superior endplate of T12 (Fig. 2). This appearance was highly suggestive of septic spondylodiscitis. Magnetic resonance imaging (MRI) findings were typical of spondylodiscitis. Gadolinium-enhanced T1-weighted MRI indicated increased signal intensity at T11–T12 vertebral bodies and severe cord compression and epidural abscess at T11–T12 associated with infiltration of soft paravertebral tissues (Fig. 3). Laboratory findings showed C-reactive protein of 0.51 mg/dl and a white blood cell count of 6100/ul. The result of the patient’s blood culture was negative. On the seventh day after readmission, he underwent partial laminectomy at T11 and posterior fusion at T9–L2. *A. terreus* was isolated from the material of T11–T12 intervertebral disc and vertebral bodies. His *Aspergillus* antigen was positive in the blood examination. The operative specimen from the T12 vertebra revealed inflammatory granulation between trabecular bone and the presence of numerous neutrophils in the granuloma. Histological examination showed chronic suppurative osteomyelitis (Fig. 4). On the 35th day after admission, he underwent anterior fusion at T11 and T12 with a rib bone graft (Fig. 5). Voriconazole (VRCZ) 600 mg intravenous was administered for 2 months, and VRCZ 600 mg oral was subsequently administered for 3 months. The patient wore a rigid corset for 5 months. Posterior partial laminectomy at T11 and anterior fusion at T11 and T12 resulted in a good clinical course. The patient’s neurological dysfunction was completely recovered, and his back pain had disappeared. Two years after the operation, CT showed bone fusion at T11 and T12 (Fig. 6). MRI revealed no evidence of increased signal intensity at T11–T12 vertebral bodies and severe cord compression and epidural abscess at T11–T12 (Fig. 7).

## Discussion

*A. terreus*, a saprophytic fungus, is recognized as an emerging pathogen in various infections in humans. However, bone and joint involvement is uncommon [1]. Bone aspergillosis is a rare disease, accounting for 1.8% of aspergillosis cases [2]. Molds belonging to the genus *Aspergillus* are important causes of fungal spondylodiscitis. *A. fumigatus*, *A. flavus*, and *A. nidulans* are the most commonly isolated species [3], and there are only a few reports of vertebral infections involving *A. terreus*. Mechanisms involved in *A. terreus* spondylodiscitis include contiguous spread from adjacent pulmonary foci; hematogenous dissemination; and, more rarely, direct inoculation (for example, trauma or surgery) [4]. We describe a 74-year-old Japanese man with a rare case of *A. terreus* spondylodiscitis after an abdominal penetrating injury.

The natural ecological niche of *A. terreus* is the soil, where it survives and grows on organic debris [5]. To the best of our knowledge, only seven cases of spondylodiscitis caused by *A. terreus* have been reported previously in humans. In two cases reported by Seligsohn *et al.* [6] and Brown *et al*. [7], respectively, the infection may have resulted from hematogenous dissemination secondary to intravenous drug injection. Two other cases were also described in immunocompromised patients; however, the infection of the lumbar discs with *A. terreus* was due to contiguous spread from a primary pulmonary focus [8, 9]. Moreover, the case reported by Glotzbach *et al*. [10] was an unusual complication of an aortofemoral vascular graft. The fungus might have been acquired at the time of initial surgery followed by systemic propagation. Maman *et al*. [11] reported a case with multifocal bone involvement occurring in a seemingly immunocompetent host, without elucidation of how the disease was acquired. Comacle *et al*. [1] reported a case of an immunocompetent 20-year-old man in whom the infection possibly arose from traumatic inoculation of the fungus during a previous motorbike accident.

We report a rare case of a 74-year-old Japanese man with *A. terreus* spondylodiscitis after an abdominal penetrating injury. The patient initially sustained an anterolateral bone avulsion of the left T12 vertebral body after an abdominal penetrating injury. X-ray image showed slowly progressing spondylodiscitis. Thirteen weeks after the injury, *A. terreus* was isolated from the material of the T11–T12 intervertebral disc and vertebral bodies. The *Aspergillus* antigen was positive in the blood examination. The operative specimen in T12 vertebra revealed inflammatory granulation between trabecular bone and the presence of numerous neutrophils in the granuloma. Histological examination showed chronic suppurative osteomyelitis. These histological findings of chronic suppurative osteomyelitis and the radiological findings strongly suggested direct inoculation of *A. terreus*. The presence of *A. terreus* on the L-shaped metal peg while it was on the ground directly may have resulted in inoculation at T11 and T12 during penetration. However, it is not possible to completely rule out the possibility of hematogenous dissemination.

Sharp-force injuries are relatively frequent in urban violence. They are well-documented as a cause of death, representing the second leading cause of death by homicide in the United States and the primary cause in Europe, Africa, and Asia. Epidemiological data of the living victims are sparse, but the consequences and complications of these injuries are well-known. In particular, infection is the leading complication of penetrating abdominal trauma. Infections occur in 10–15% of cases and mainly include abscesses, peritonitis, and gaseous gangrene [12, 13]. We found two reports of *Candida albicans* spondylodiscitis after an abdominal penetrating injury [14, 15]. No report of spondylodiscitis due to *A. terreus* after an abdominal penetrating injury was noted.

## Conclusions

To our knowledge, this is the first report on spondylodiscitis caused by *A. terreus* after an abdominal penetrating injury. The histological examination of chronic suppurative osteomyelitis and the radiological findings strongly suggested direct inoculation of *A. terreus*.

## Abbreviations

CT: Computed tomography
MRI: Magnetic resonance imaging
VRCZ: Voriconazole
